# Molecular and Electronic Structures of Neutral Polynitrogens: Review on the Theory and Experiment in 21st Century

**DOI:** 10.3390/ijms23052841

**Published:** 2022-03-04

**Authors:** Oleg V. Mikhailov

**Affiliations:** Department of Analytical Chemistry, Certification and Quality Management, Kazan National Research Technological University, K. Marx Street 68, 420015 Kazan, Russia; olegmkhlv@gmail.com

**Keywords:** neutral polynitrogen, molecular structure, stability, quantum chemical modeling

## Abstract

The data on the existence and physicochemical characteristics of uncharged single element chemical compounds formed by nitrogen atoms and containing more than two nuclides of this element (from N_4_ to N_120_, oligomeric and polymeric polynitrogens) have been systematized and generalized. It has been noticed that these data have a predominantly predictive character and were obtained mainly using quantum chemical calculations of various levels (HF, DFT, MP, CCSD etc.). The possibility of the practical application of these single element compounds has been considered. The review mainly covers articles published in the last 25 years. The bibliography contains 128 references.

## 1. Introduction

Nitrogen is known to be one of the most important chemical elements, due to its already predominant concentration in the atmosphere of our planet (78.3% of its volume). In addition, this element is a key component of proteins and polypeptides. In the Earth’s atmosphere, nitrogen exists in the form of a simple substance, namely, dinitrogen, which has the chemical formula N_2_. Although dinitrogen was first isolated from atmospheric air almost 250 years ago (1772), independently, by Rutherford, Scheele and Cavendish, this compound, in fact, still remains the sole stable simple substance formed by the atoms of the given chemical element. In the given compound, nitrogen atoms are bonded to each other by an N≡N triple bond; owing to this, the dinitrogen molecule is characterized by both a very high dissociation energy (945.41 kJ/mol) and a very low chemical activity [1,2]. It should be noted that, when a triple bond is broken, most of the energy of this bond (about 58%) is spent on breaking just the first of the components of this bond [3,4]. It determines the resistance of the dinitrogen molecule to those reagents that, upon breaking one nitrogen π-bond, form one or two bonds with the nitrogen atom. That is why, in particular, the H_2_ molecule and even atomic hydrogen (H), under normal conditions, are not able to interact with N_2_: the formation of one (when an H atom is attached) or even two (when an H_2_ molecule is attached) N–H bonds do not compensate the energy spent on breaking π-bonds in the dinitrogen molecule (nor H–H bonds, in the case of addition of H_2_) and these reactions, accompanied by the formation of the •N_2_H radical and the diimine H_2_N_2_ molecule, respectively, are very energy intensive. Nevertheless, after the breaking of the first π-bond in the N_2_ molecule and the formation of any of the above mentioned chemical compounds, further breaking of each of the remaining two bonds requires much less energy. When three H_2_ molecules are attached to an N_2_ molecule, the energy of the resulting N–H bonds compensates the energy expended on breaking the N≡N bond and the formation of ammonia NH_3_, as a result, turns out to be an exothermic process. At a temperature of T < 63.29 K and a pressure of 101,325 Pa, dinitrogen is in a solid aggregate state, and three modifications are currently known for it: α-N_2_ with a cubic lattice, space group *P213* and period a = 5.660 Å, existing at temperatures below 36.61K; β-N_2_ with hexagonal close packing, space group P63/mmc and lattice parameters a = 3.93, c = 6.50 Å, existing in the temperature range 36.61–63.29 K; and γ-N_2_, arising at pressures above 350 MPa and temperatures below 83.0 K, with a tetragonal crystal lattice [1]. In principle, they may be considered as allotropic modifications of nitrogen. In addition to dinitrogen, the existence of only one neutral simple substance formed by nitrogen atoms, namely, tetranitrogen N_4_, has been experimentally proven with a sufficient degree of reliability [5,6].

Today, the total number of works devoted to single element polynuclear nitrogen compounds—the socalled polynitrogens—already measures a three digit number. During different years, quite detailed review articles have been published on this subject (in particular, [7,8,9]). However, all these reviews were published more than 10 years ago, according to original works that were published before the beginning of the 21st century. At the same time, in each of these reviews, the literature data systematics and discussion were based on the number of atoms in the molecules of polynitrogens. Such a systematic of neutral polynitrogens seems quite justified; taking into account all of the above, this review is devoted mainly to the consideration of those works on single element polynuclear nitrogen compounds that have appeared in the current 21st century.

## 2. Theoretical Background for the Existence of Various Polynitrogens

For the formation of polynuclear compounds with E–E bonds (E—an atom of any chemical element) of any multiplicity, the ability of atoms of a given element to form homochain structures with a sufficiently large number of atoms is necessary. The most desirable way is when the atoms of the element E have the same number of valence orbitals, number of valence electrons, and the maximum possible coordination number. Such a coincidence, however, occurs only for one chemical element, namely, carbon; in the case of nitrogen, where these numbers are four, five, and four, respectively, such complete coincidence is absent. Nevertheless, more than one or two chemical compounds, in which three or more atoms of a given element are interconnected, are known; this fact gives us hope for the existence and possibility of obtaining a wide variety of polynitrogens. However, it can be assumed that, since the nitrogen atom in its chemical compounds, as a rule, forms no more than three chemical bonds with other atoms by the exchange mechanism, this will occur for only those that contain either groups of atoms (–N=N–) or (>N–N<) groupings, or both taken together. From the standpoint of classical ideas about the valence capabilities of chemical elements, including such chemical bonds that a nitrogen atom usually forms with other elements (namely, three), the total number of nitrogen atoms in any stable neutral polynitrogen should be an even number. On the other hand, in this variant, any of the stable neutral polynitrogens, according to the classical concepts, should have a closed (cyclic) structure, because, otherwise, it is impossible to ensure that each nitrogen atom in the polynitrogen would form, namely, three—not more or less—chemical bonds with their neighbors. It does not exclude the possibility of the formation of such polynitrogens, in which individual nitrogen atoms form more than three (namely, four or even five) chemical bonds with their neighbors (for example, in the case of the diazide N≡N=N–N=N≡N). Moreover, it is possible that their stability will be even higher than the stability of polynitrogens with the same number of nitrogen atoms, but with a closed loop of nitrogen atoms. Closed geometric structures with nitrogen atoms at their vertices can be either two dimensional or three dimensional, or a combination of these. Potential two dimensional structures for polynitrogens can be individual polygons with an even number of vertices, as well as their various combinations with a total even number of vertices (in this case, no more than three sides should converge at each of the vertices). Potential three dimensional structures can be various polyhedra, but, in this case, at any vertex, with the valence possibilities of nitrogen atoms, should be three valence angles—no more and no less. These polyhedra include three of the five Platonic solids, namely, the regular tetrahedron, cube and regular dodecahedron; seven of the thirteen Archimedian solids (truncated tetrahedron, truncated octahedron, truncated cube, truncated cuboctahedron, truncated dodecahedron, truncated icosahedron, rhombic truncated icosidodecahedron); four of the five Fedorov solids (hexagonal prism, rhombic dodecahedron, as well as the already mentioned cube and truncated octahedron) and many other less symmetrical polyhedra (in particular, polygonal prisms with an even number of vertices) satisfying the above condition. Taking into account the above, first of all we will consider the literature data array related to neutral polynitrogens with an even number of atoms.

## 3. Neutral Polynitrogens N_k_ Containing an Even Number of Atoms

With some degree of conventionality, these polynitrogens, depending on the number of atoms in the structural unit, can be divided into three categories: those with the number of atoms from 4 to 10, with the number of atoms from 12 to 20, and with the number of atoms more than 20. We will consider these categories in this order.

### 3.1. Polynitrogens N_4_–N_10_

**Scheme 1:**
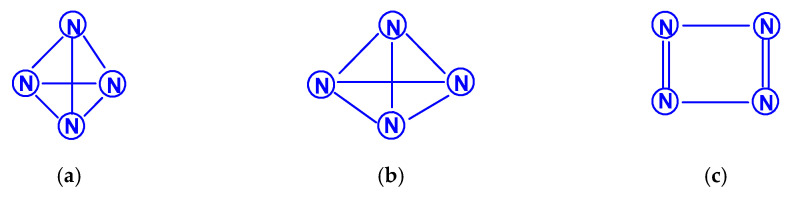
(**a**)**:** N_4_ (*T_d_*); (**b**)**:** N_4_ (*D_2d_*); (**c**)**:** N_4_ (*D_2h_*).

**Scheme 2:**
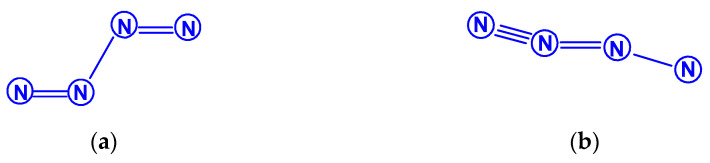
(**a**)**:** N_4_ (*C_2h_*); (**b**)**:** N_4_ (*C_s_*).

For tetranitrogen N_4_, the existence of three isomeric forms can be expected, in each of them, all nitrogen atoms are trivalent, namely, tetrahedral (*T_d_* symmetry), distorted tetrahedral (*D_2d_* symmetry) with three N–N single bonds, and rectangular (*D_2h_* symmetry) with two single bonds and two N=N double bonds. The images of their molecular structures are presented in Scheme 1. Isomers with a different shape, namely, zigzag or with two double N=N and one single N–N bonds (*C_2h_* symmetry), or with one triple N≡N, one double N=N and one single N–N bonds (*C_s_* symmetry) (Scheme 2) are also not excluded. This chemical compound of nitrogen was first mentioned in [10], where, however, it was postulated as a “van der Waals complex”, i.e., (N_2_)_2_, in which N_2_ dinitrogen molecules are interconnected due to only dispersion forces. In later works [11,12,13,14,15,16,17,18,19,20,21,22,23,24], the possibility of the existence of isomeric tetranitrogens was theoretically confirmed by quantum chemical calculations using quantum chemical methods at various levels; moreover, in most of these works, methods with the functionals Hartree–Fock (HF) theory, perturbation theory (MP), quadratic configuration interaction with singles and doubles (QCISD) and the theory of linked clusters (CCSD and CCSD(T)) [11,15,18,19,22,23] were used, and only in [12,14,15,17,24], various methods of lower levels, in particular density functional (DFT).

Literature data on the relative stability of these isomers diverge quite noticeably from each other. Thus, according to the calculations performed in [12] by the MBPT2 method, the N_4_ (*D_2h_*) isomer has a total energy 12 kcal/mol higher than the N_4_ (*T_d_*) isomer, while the calculations by the QCISD(T)/6-311 + G* [13] gave the opposite result (lower by 3 kcal/mol). According to one of the recent works [22], using the QCISD(T)/TZVP method to calculate the molecules of these two isomers, the difference in total energy between the tetrahedral and rectangular isomers is very small and amounts to only 0.50 kJ/mol, i.e., these isomers are energetically equivalent. Summarizing the presented data, as well as other theoretical studies of tetranitrogens [16,19,20,23,24], it can be argued that the rectangular shape of N_4_ is more stable. A detailed calculation of the parameters of the molecular structures (bond lengths, bond and torsion angles) of these two theoretically expected forms of tetranitrogen was carried out in [23,24].

The existence of the “rectangular” isomer N_4_ (*D_2h_*) was noted by experiment in [5,6,19]. It was obtained from the N_4_^+^ cation and was found in the gas phase; in experiments based on neutralization reionization mass spectrometry, the lifetime of its molecule was more than 1 μs; therefore, it is predicted to be a metastable compound. Bittererová et al. [16] used the CASSCF method to study the dissociation of this isomer, as a result, a new transition state with *C_2v_* symmetry was discovered with an effective dissociation barrier of 6.5 kcal/mol. Tetrahedral tetranitrogen N_4_ (*T_d_*) was studied in detail in [11,15,18], but this isomer has not yet been reliably found experimentally. In this regard, it is interesting to note that, according to the data of [18], the most energetically stable is not N_4_ (*T_d_*) or N_4_ (*D_2h_*), but azidonitrene N≡N=N–N, the triplet ground state of which (^3^A”) within RCCSD(T)/6-311+G(3df) and MRCISD+Q/6-311+G(3df)) methods confirmed to be 56 kJ/mol lower than the tetrahedral *T_d_* isomer (the ground state of which is a spin singlet).

**Scheme 3:**
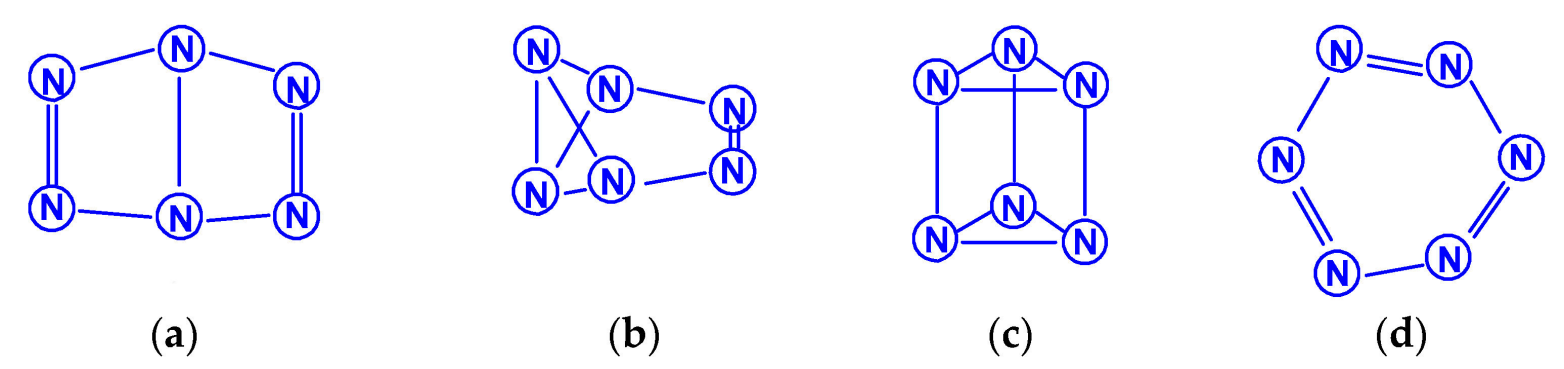
(**a**): N_6_ (*C_2v_*); (**b**): N_6_ (*C_2v_*); (**c**): N_6_ (*D_3h_*); (**d**): N_6_ (*D_3h_*).

**Scheme 4:**
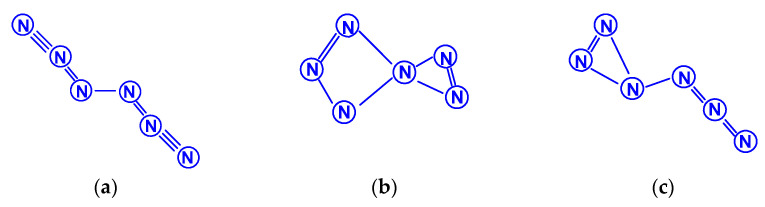
(**a**): N_6_ (*C_2h_*); (**b**): N_6_ (*C_2v_*); (**c**): N_6_ (*C_s_*).

**Scheme 5:**
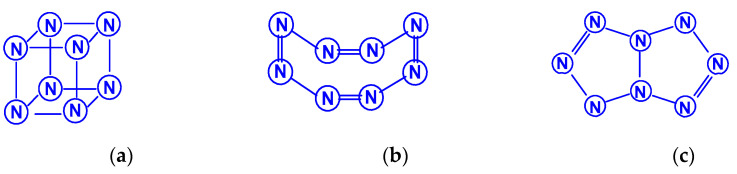
(**a**): N_8_ (*O_h_*); (**b**): N_8_ (*D_2d_*); (**c**) N_8_ (*D_2h_*).

**Scheme 6:**
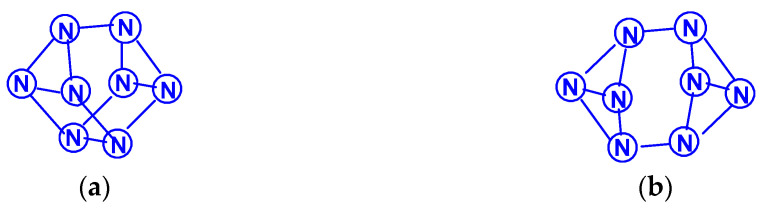
(**a**): N_8_ (*C_2v_*); (**b**) N_8_ (*D_2h_*).

The first report on the next most complex category of polynitrogens with even k, namely, hexanitrogens, appeared more than 50 years ago in [25]. In this work, the formation of N_6_ was suggested as a result of the dimerization of N_3_ radicals formed during the interaction between the azide anion N_3_^−^ and the hydroxyl radical. The nature of this compound was not identified with sufficient reliability. Theoretically, for hexanitrogens, the presence of at least five isomeric forms can be expected, in each of which all nitrogen atoms are trivalent, namely, the “open book” N_6_ (*C_2v_*) with two double bonds (an analog of dewarbenzene, hexaazabicyclo[2.2.0]hexadiene-2,5), hexaazatricyclo [3.1.0.0]hexene-3 (analogue of benzvalene) N_6_ (*C_s_*), trigonal prism (hexaazatetracyclo[2.2.0.02.6.03.5] hexane, hexaazaprismane) N_6_ (*D_3h_*) and “twisted boat” N_6_ (*D_2_*) [9], as well as a planar hexagon with three conjugated double bonds (hexaazabenzene, hexaazine) N_6_ (*D_3h_*) (Scheme 3). Assuming the possibility of the presence, in N6, of nitrogen atoms with a different number of chemical bonds formed by them (for example, two, four, and five), the assortment of possible hexanitrogens will expand significantly; the simplest of them is diazide N6 (C2h) (N≡N=N–N=N≡N), the formation of which, very likely, was observed in [25], indicated above, and, also, two structures with *C_2v_* and *C_s_* symmetry (Scheme 4). In later theoretical works devoted to N_6_ [12,14,26,27,28,29,30,31,32,33,34,35], however, attention was paid to the fact that the shape of the potential energy surface depends very strongly on the calculation method used. Therefore, hexagonal molecular structures (hexaazabenzene is not among those) and N6 molecular structures were identified as stable minima on the potential energy surface in the case of using the Hartree–Fock and MP2 methods [14,35], DFT with a B3PW91 functional and CCSD (T) [36]. The existence of N_6_ molecules with prismane, dewarbenzene, benzvalene and “twisted boat” structures are predicted by these theoretical methods. The systematic study of hexanitrogens using various methods of quantum chemistry began only with the onset of the 21st century, which resulted in the appearance of works [37,38,39,40,41,42,43,44,45]. These cyclic isomers of N_6_, the existence of which was confirmed in the works of the 20th century, as well as the acyclic isomer, diazide, were considered in [37,38,39,40,41]. In the earliest chronologically [37], the process of diazide destruction according to the N_6_ → 3N_2_ general scheme was investigated in the framework of the second order multiconfiguration perturbation theory CASSCF/CASPT2, in combination with a preliminary study of this process by the DFT method. In this case, a transition state with *C_2_* symmetry was found between the most stable linear diazide form of N_6_ and three N_2_ molecules, with an energy barrier of 28.7 kcal/mol according to the CASSCF/CASPT2 method and 17.4 kcal/mol according to the DFT method, respectively. A similar decomposition process was also discovered in [38,39,40,41]; in [40,41], in addition to the process of the decomposition of diazide, the processes of the decomposition of other isomeric N_6_ molecules with the formation of molecular dinitrogen, namely, “twisted boat” and hexaazabicyclo[2.2.0]hexadiene-2,5, were also analyzed, and the molecular structures of these three compounds were calculated. According to these results, the most stable of these three isomers is diazide, while N_6_ (*C_2v_*) and N_6_ (*D_2_*) isomers have higher total energies than diazide (by 26.2 and 62.5 kcal/mol, respectively). In this case, the N_6_ (*D_2_*) isomer decomposes in one step with a very low activation barrier, while the decomposition of N_6_ (*C_2v_*) occurs by a stepwise mechanism [41]. The transformation of prismatic N_6_ (*D_3h_*) into N_2_, as well as the analogous process for N_6_ (*D_2_*), is also a one stage process and is characterized by a low energy barrier [40,42]. It was shown in [43] that chain, ring, framework, and prismatic framework nitrogen-containing molecular structures can be clearly separated by energy capacity. In particular, the prismatic molecule N_6_ (*D_3h_*) is noted to have a very high dissociation barrier to N_2_ and, if it could be synthesized, it would be very stable (allowing storage at ambient conditions). During the reaction of its dissociation, a very large amount of energy is released, and without the formation of any harmful substances [43]. In some works, devoted to hexanitrogens, namely, [44,45,46], the possibility of the existence of hexaazabenzene (hexaazine) N6 was analyzed. It was shown in [44] that the planar cyclic structure of N_6_, which is isoelectronic to benzene, can be sufficiently stable if “peripheral” oxygen atoms are added to it, bound to nitrogen atoms by covalent or coordination bonds. Another potential possibility of stabilizing such a structure was proposed in [45], where a theoretical consideration was carried out of the stabilization of the particles of the cyclic N_6_ fragment in the form of a planar hexagonal ring in systems M(η6 -N_6_), where M = Ti, Zr, Hf or Th. It was found that these systems have total energy minima at the N_6_ (*C_6v_*) geometry that corresponds to the η6 complex of the corresponding M with the planar hexaazine ring N_6_, which is 70–130 kcal/mol higher than the energy of the metal atom and three N_2_ molecules, but have significantly lower energy of any of the N_6_ isomers. It was suggested that the stabilization of the N6 planar structure is associated with the interaction of the ring π-system with d- (in the case of M=Ti, Zr, Hf) or f- (M=Th) orbitals; in such a case, five chemical bonds are formed between the N_6_ and M rings due to the donor–acceptor (dative) mechanism. In [46], using the HF, MP2, and DFT methods, the structure of hexaazine and its monoprotonated (N_6_H^+^) was discussed and it was shown that, for N_6_ molecules, the so called HOMED index (electron delocalization harmonic oscillator model), based on the extended geometry, which is a measure of p-electron delocalization, is close to the HOMED index of the benzene molecule. The protonation of one N atom in planar N_6_ does not significantly affect the HOMED value, and, unlike the protonated benzene molecule, planar N_6_H^+^, like the original N_6_ (*D_3h_*), is an aromatic compound; wherein, the p-electrons of the N_6_ ring do not participate in the formation of the N–H bond. Nevertheless, the data of recently published works [22,23], where the QISCD(T)/TZVP method was used for the calculation, clearly indicate, on the one hand, the impossibility of realizing the isolated N_6_ (*D_3h_*) molecule, and, on the other hand, that the most stable among all the cyclic modifications of N_6_, is, namely, N_6_ (*C_2v_*) (hexaazabicyclo[2.2.0]hexadiene-2,5). Reliable information about obtaining any hexanitrogens in the experiment (with the possible exception of diazide, which, judging by the data of various quantum chemical calculation methods, has the lowest total energy compared to other hexanitrogens), is currently not available in the literature. A fairly significant number of studies are devoted to octanitrogens, however, almost all the results and conclusions presented in them are based on theoretical data, revealed mainly by quantum chemical calculations. One of the first (and, perhaps, the very first) works devoted to octanitrogens is the work [47], published more than 40 years ago; it was shown by the SCF pseudopotential method that octaazacubane (cubic N_8_, *O_h_* symmetry) has higher energy than two N_4_ molecules, and, consequently, its independent existence becomes problematic. Nevertheless, taking into account the high symmetry of this structure, it can be expected, according to [9], that N_8_ (*O_h_*) should have a fairly significant activation barrier for its decomposition to form dinitrogen N_2_. In later publications [48,49,50,51,52,53,54,55,56,57], a number of other isomeric forms of N_8_, among which there are both cyclic and acyclic structures, as well as “hybrid” structures containing both of these components, were analyzed. For example, the authors of the article [48] also used ab initio methods of molecular electronic structure to study, in addition to N_8_ (*O_h_*), two more N_8_ isomers, namely, N_8_ (*D_2d_*), similar to cyclooctatetraene (octaazacyclooctatetraene), and N_8_ (*D_2h_*), which has a planar bicyclic form (octaazapentalene) (Scheme 5). These optimized geometries of various N_8_ molecules were obtained using DZP basis sets and SCF, MP2, CISD and CCSD methods. Their vibrational analysis indicates that all three structures represent potential energy minima with the octaazapentalene bicyclic structure being the absolute minimum (225 kcal/mol above four N_2_ molecules) N_8_ (*O_h_*) at 198 kcal/mol, while N_8_ (*D_2d_*) is only 35 kcal mol/1 higher than azapentalene. The same results are confirmed in [49]. In [50], two novel structures were added to these structures: N_8_ (*D_2v_*), designated as octaazacuneane, and N_8_ with the same *D_2h_* symmetry as in octaazapentalene (Scheme 6), but, unlike octaazapentalene, it lacks N=N double bonds (it can be considered as a combination of two tetrahedra connected to each other by two N–N single bonds). In this work, nine (!) quantum chemical methods of various levels were used for the calculation, the data of which, both in terms of the structural and energy parameters of the N_8_ molecules under consideration, are in fairly good agreement with each other (at least in a qualitative sense). In addition, it was shown, on the one hand, that both of these isomers have minima on the N_8_ potential energy hypersurface, and, on the other hand, that they have a higher energy than the N_8_ isomers containing N=N double bonds. Among the three N_8_ isomers considered in [50], which have only single bonds, those with higher symmetry have higher total energies: for example, according to the CCSD(T)/DZP method, the total energies of N_8_ (*D_2h_*) and N_8_ (*C_2v_*) are less than the total energy of N_8_ (*O_h_*) by 35.7 and 65.1 kcal/mol, respectively. In later work [51], along with octaazapentalene, two more N_8_ isomers, azidopentazole and diazidodiimide, were described using ab initio calculations, and, also, the potential energy surfaces and the transition structures’ nature during the decay of each of these three N_8_ isomers into dinitrogen N_2_ were studied. All these isomers are high energy species compared to molecular nitrogen, but their energy is much lower than that of the previously studied cubic structure N_8_ (*O_h_*). According to the data of perturbation theory of second order (MP2), the dissociation of octaazapentalene proceeds via isomerization to a linear molecule. The dissociation reaction of azidopentazole prefers ring scission with a cost of less than 20 kcal/mol, over bond scission in the side chain. The diazidodiimide cis-isomer was proven to be slightly more stable than the trans-isomer, at the highest levels of the theory used here. In [14,42,52,53,54,55], the other N_8_ isomers were also described, so that the total number of considered compounds with a given stoichiometric composition is currently measured at several dozen. There are also quasi-ionic compounds among them, for example, N_5_^+^N_3_^−^,predicted in [55] using ab initio methods. In [56], the structure of a solid with a molecular lattice, consisting of N_8_ molecules, was calculated, and a possible obtaining way was proposed.

The possibility of synthesizing any of the octanitrogens in the experiment with its subsequent isolation from the reaction system remains open. It should be noted that, in a recently published work [57] using hydrazinium azide [N_2_H_5_]^+^(N_3_)^−^, the N_8_ isomer with the structure represented as (N≡N^+^)–(N^−^)–N=N–(^−^N)–(^+^N≡N), was obtained. It was shown, using the methods of confocal micro-Raman spectroscopy and synchrotron powder X-ray diffraction, that at 13–15 GPa [N_2_H_5_]^+^(N_3_)^−^ passes into a new form without changing its composition, the formation of which is associated with the internal strengthening of hydrogen bonds, and with an increase in pressure up to 40 GPa—is transformed into N_8_. This compound formation is accompanied by the emergence of new peaks at 2384, 1665, and 1165 cm^−1^, arising from the terminal N≡N stretching, the central N=N stretching, and the N–N stretching, respectively. This compound is resistant to degradation to N_2_ at pressures above 25 GPa and decomposes completely at pressures below 3 GPa. There are no other similar reports, apart from the paper just cited [57].

**Scheme 7:**
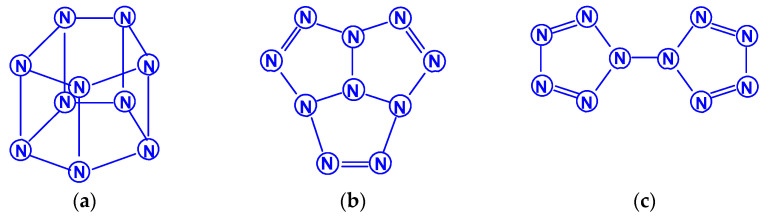
(**a**) N_10_ (*D_5h_*); (**b**) N_10_ (*D_3h_*); (**c**)N_10_ (*D_2h_*).

**Scheme 8:**
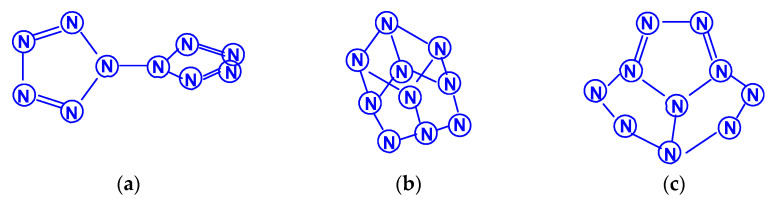
(**a**): N_10_ (*D_2d_*); (**b**): N_10_ (*C_3vm_*); (**c**) N_10_ (*C_3vc_*).

**Scheme 9:**
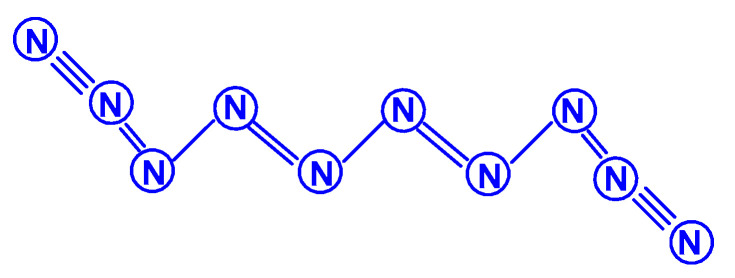
N_10_ (*C_2h_*).

The study of decanitrogens began in [58]. In this publication, six possible N_10_ isomers were considered: in the form of a pentagonal prism N_10_ (*D_5h_*); three articulated pentagons N_10_ (*D_3h_*); two pentagons; two vertices that are connected to each other, and which are themselves are in the same plane N_10_ (*D_2h_*) (Scheme 7); two pentagons, two vertices of which are connected to each other and are in mutually perpendicular planes N_10_ (*D_2d_*); as well as two more complex structures N_10_ (*C_3vm_*) and N_10_ (*C_3vc_*) (Scheme 8). Using the DFT B3LYP/6-31G* methods and the MP2/6-31* perturbation theory with the combination of the Gaussian94 program, geometric optimization of the structures was carried out and their vibration frequencies and thermochemical parameters were calculated. According to the data of this work, the total energies of the above isomers (*E_total_*) correlate with each other as *E_total_* [N_10_ (*D_2d_*)] < *E_total_* [N_10_ (*D_2h_*)] < *E_total_* [N_10_ (*C_3vm_*)] < *E_total_* [N_10_ (*D_3h_*)] < *E_total_* [N_10_ (*C_3vc_*)] < *E_total_* [N_10_ (*D_5h_*)], i.e., the isomer with the lowest symmetry is the most stable. It is interesting that N_10_ (*D_3h_*) with a flat structure of fused triple five membered rings is a transition state for the N_10_ (*C_3v_*) molecule, with the trigonal–pyramidal structure. Since the publication of this work, only a few works devoted to N_10_ have appeared, namely, [59,60,61,62,63,64], as well as the recent publications mentioned above [42,43]. Therefore, in [59], the N_10_ (*D_2h_*) and N_10_ (*D_2d_*) isomers were studied using the HF, MP2, B3LYP, and QCISD methods, and a saddle point for N_10_ (*D_2h_*) (five membered rings on the same plane) and a local minimum for N_10_ (*D_2d_*) (two rings in two planes perpendicular to each other), separated by an energy of 3–6 kcal/mol (depending on the calculation method used), were found. The N–N bond energy between these five membered rings was estimated at 93 (QCISD) and 84 (B3LYP) kcal/mol, indicating a fairly strong bond between them. Based on this, it was suggested that N_10_ could be a promising building block for more complex polynitrogens (in particular, N_60_) [59]. In search of minima on the potential energy surface of the N_10_ molecule, nine structures without double bonds were studied in [60] and it was found that all nine are minima at RHF, eight are minima at B3LYP, and seven are minima at the level of MP2 theory. In [61], a theoretical calculation of the molecular structure for the acyclic isomer N_10_ (*C_2h_*), shown in Scheme 9, potential energy surfaces and activation energy barriers for the dissociation reaction N_10_ → N_8_ + N_2_ were performed using the HF, MP and CCSD(T) theory levels. By the CCSD(T) data, this barrier is approximately 17–18 kcal/mol; based on this, acyclic N_10_ is easily dissociated and therefore not suitable as a high energy density material. Using the DFT B3LYP and MP2 methods, the authors of [62] were able to find 11 energy minima on the potential energy surface with activation barriers of the order of 10 kcal/mol for dissociation or isomerization reactions, allowing them to suggested the kinetic instability of the N_10_ molecules, among which three are acyclic, five are monocyclic with five membered cycles, and three are bicyclic with similar cycles with a range of total energies from −547.26218 Hartree in the case of the bicyclic structure N_10_ (*D_2d_*) to −546.87618 Hartree in the case of one of the acyclic structures (i.e., relative energies from 0 to 242.2 kcal/mol). Based on later work data [63], in which the stability of nine N_10_ structures was considered at the level of the G3 theory, the most stable structure is a bowl shaped structure with three five membered rings and *C_3v_* symmetry. It should be noted that the total energy of this structure is −546.89556 Hartree, which is actually only much greater than the total energy of N_10_ (*D_2d_*) (however, such a comparison is not entirely justified due to the difference in the calculation methods for obtaining these values). The structural variety of decanitrogens is not exhausted by the N_10_ structures listed above, and it is possible that another molecular structure, even more stable than this cup shaped structure, will be found in the future. In any case, it is too early to conclude this question, especially because of a recently published work, [64]. Bipentazole N_10_ (*D_2d_*) was found to be the most stable form of decanitrogen [64], consistent with the data of the work already cited above [62].

### 3.2. Polynitrogens N_12_–N_20_

This group of polynitrogens, however, is the subject of a relatively small number of works [65,66,67,68,69,70,71,72,73,74,75,76]. Most of them relate to the 21st century; the articles [65,66] are the only exceptions. In this paragraph, they are considered in ascending order of the number of nitrogen atoms in their molecular structures.

**Scheme 10:**
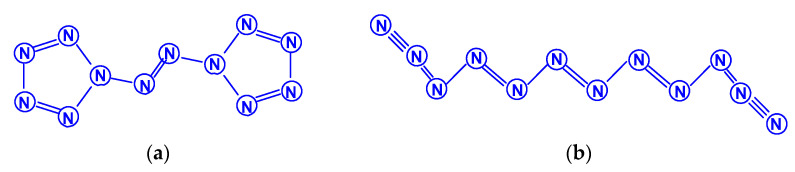
(**a**) N_12_ (*C_2h_*); (**b**) N_12_ (*C_2h_*).

**Scheme 11:**
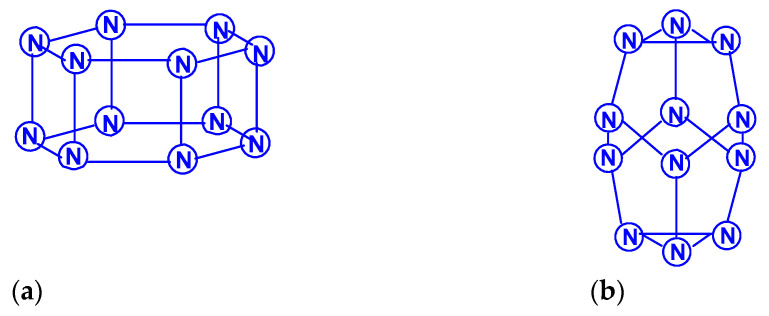
(**a**): N_12_ (*D_6h_*); (**b**) N_12_ (*D_3d_*).

**Scheme 12:**
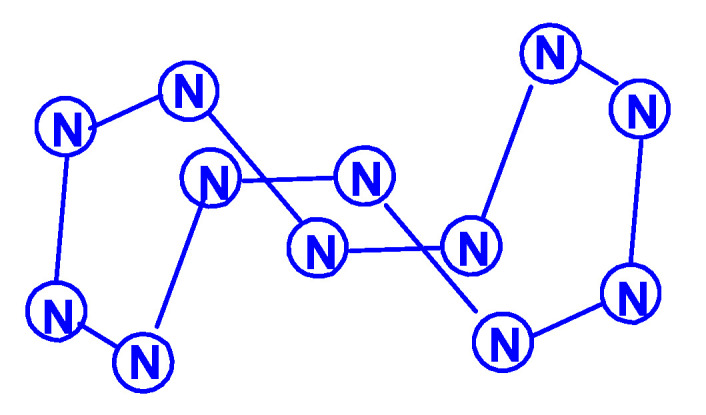
N_12_ (*D_3d_*).

Dodecanitrogens N_12_ were studied in the works [65,66,67,68,69]. In the earliest work [65], two isomeric compounds were considered, namely, diazobispentazole N_12_ (*C_2h_*), whose structure contains two pentazole fragments linked by an azo group (–N=N–), and a planar acyclic isomer N_12_ with the same symmetry (Scheme 10). Their calculation by the DFT method showed the significantly higher stability of the first of these isomers compared to the second. In 1997, the authors of [66] theoretically studied two more N_12_ isomers: in the form of a regular hexagonal prism N_12_ (*D_6h_*) and the form of an octahedron with two triangular and six pentagonal faces N_12_ (*D_3d_*), containing only single N–N bonds, and found that there are acceptable minima on the potential energy surface of N_12_ (Scheme 11). The data presented give an opportunity to draw a reliable conclusion, that the aromatic pentazole ring is a stable structural unit for at least some larger polynitrogens with an even number of nitrogen atoms. The studies started in [65] were continued in the work [67] at the levels of the theory HF/6-31G(d) and electron correlated MP2/6-311G(d), where the transformation of diazobispentazole N_12_ (*C_2h_*) into pentaazopentazole N_10_ with *C_1_* symmetry and into azidopentazole N_8_ with *C_s_* symmetry was discovered; in this case, the transition states corresponding to these processes were also calculated. In the following, chronologically, work [68], using two DFT methods, the existence of a new isomer, N_12_, was revealed, in addition to the five isomers studied in [65,66,67], namely, N_12_ (*D_3d_*) in the form of a noncoplanar 12-gon (Scheme 12). The total energies comparison, as well as other parameters of all these six compounds, testify, for [68], to the conclusion that the most stable of them, according to the DFT B3LYP/6-311 + G(3df,2p)//B3LYP/6-31G* data, is diazobispentazole N_12_ (*C_2h_*), with a total energy of −656.91946 Hartree, and the least stable is prismatic N_12_ (*D_6h_*), with a total energy of −656.36743 Hartree (i.e., the difference in total energies between them is no less than 346.4 kcal/mol). A similar result is also confirmed using the simpler B3LYP/6-31G* method [68]. At the same time, the dissociation study of all these isomers showed that, for five out of six of them, the energy barriers of this process are less than 10 kcal/mol; the only exception is the open chain acyclic isomer with a value estimated slightly higher (14.5 kcal/mol). In any case, all of these values are very low to be considered sufficiently kinetically stable. Concluding the story about dodecanitrogens, the work entitled “What Makes an N_12_ Cage Stable?”, suggested that cyclic N_5_ fragments have a great influence on the stabilization of N_12_ polynitrogens, while cyclic N_3_ fragments have a somewhat smaller effect, which must be noted [69].

**Scheme 13:**
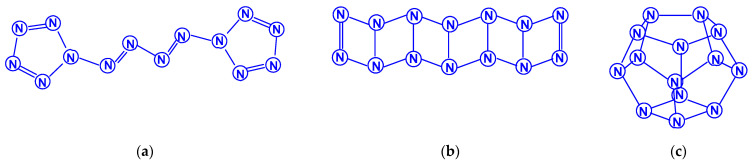
(**a**): N_14_ (*C_2h_*); (**b**): N_14_ (*C_2v_*); (**c**): N_14_ (*D_3h_*).

**Scheme 14:**
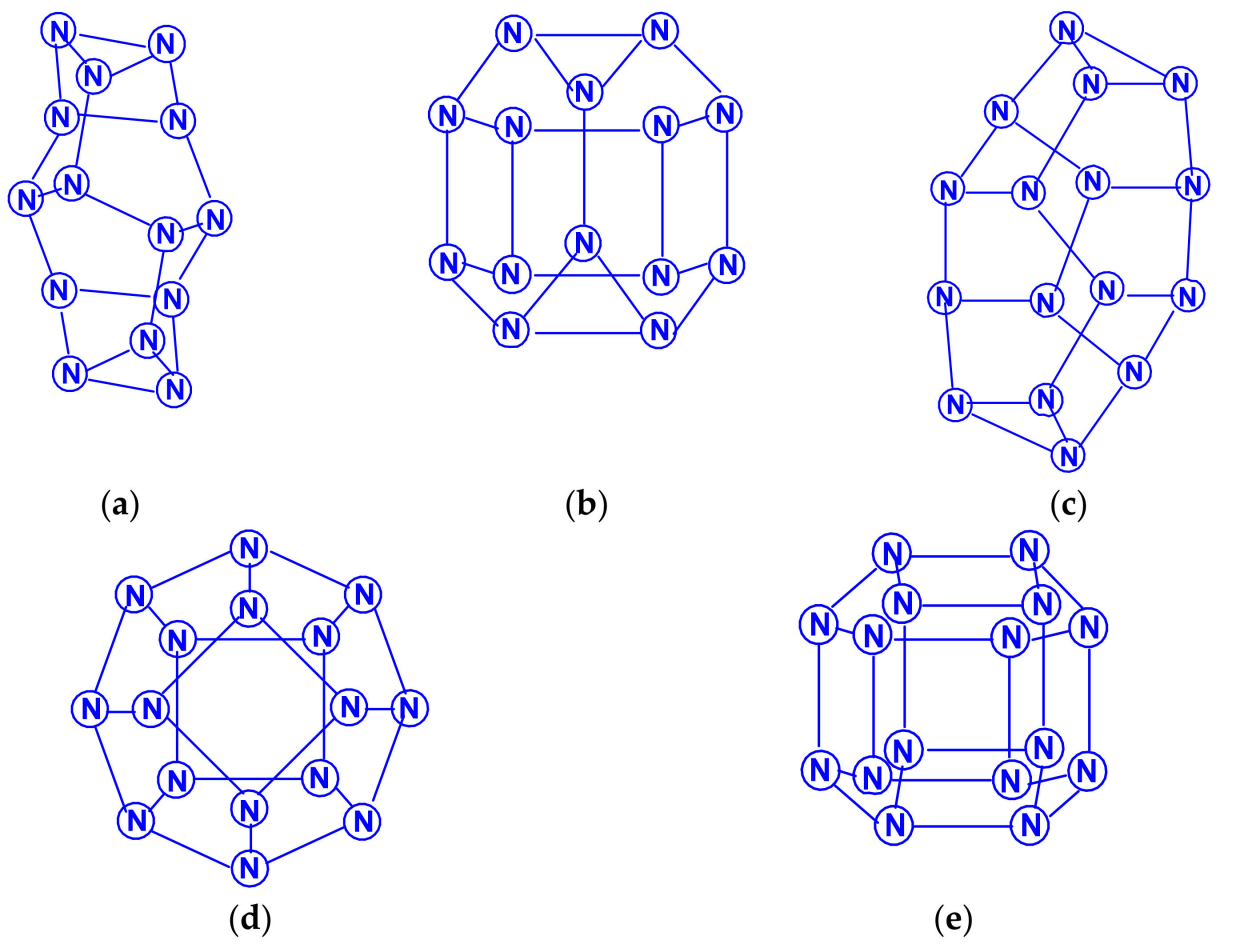
(**a**): N_14_ (*C_2_*); (**b**): N_14_ (*C_2v_*); (**c**): N_16_ (*C_2_*); (**d**): N_16_ (*D_4h_*); (**e**): N_16_ (*D_2h_*).

**Scheme 15:**
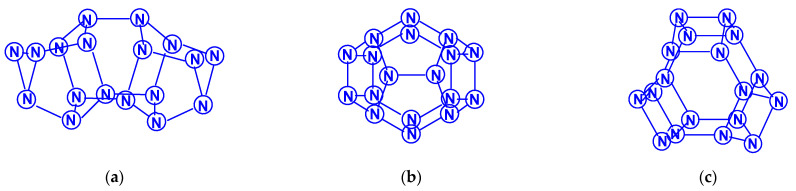
(**a**): N_18_ (*D_3h_*); (**b**): N_18_ (*C_2v_*); (**c**): N_18_ (*D_3h_*).

Only works [70,71,72,73] are devoted to polynitrogens with the number of atoms 14, 16, and 18. The first [70] considered 12 isomers of tetradecanitrogens N_14_, the majority of which contain a cyclic pentaazole fragment in their composition. The lowest energy of them is N_14_ (*C_2h_*), where two pentazole fragments are interconnected by a grouping (–N=N–N=N–), the highest energy is N_14_ (*D_3h_*) in the form of a nine-hedron with six pentagonal and three quadrangular faces; the difference in their total energy, judging by the presented data, is 397.1 kcal/mol. The plane isomer N_14_ (*C_2v_*) is intermediate in total energy (Scheme 13). In [71], 10 more isomeric tetradecanitrogens were considered, which, unlike most of those studied in [70], had a three dimensional spatial structure; among them was the isomer N_14_ (*D_3h_*), mentioned above. Interestingly, in terms of its energy stability, it won third place, with the difference in total energy between it and the most stable of these ten isomers [N_14_ (*C_3v_*)] with three triangular, three pentagonal, and three hexagonal faces) (Scheme 14) according to the HF, MP4 methods//HF and MP4//B3LYP are only 5.2, 9.1, and 10.1 kcal/mol, respectively. The least stable was the N_14_ (*C_2v_*) isomer with two triangular, three quadrangular, and four hexagonal faces, the total energy of which is higher than the total energy of N_14_ (*D_3h_*) by 87.2 kcal/mol [71]. In the article [71], along with compounds N_14_, nine spatial isomers of N_16_ (hexadecanitrogens) were also analyzed: N_16_ (*C_2_*) in the form of a decahedron with two triangular, six pentagonal, and two hexagonal faces turned out to be the most stable, and the least stable was N_16_ (*D_2h_*) with six quadrangular and four hexagonal faces, the total energy of which, according to the HF, MP4//HF, and MP4//B3LYP methods, is higher than that for N_16_ (*C_2_*) by 135.2, 135.9, and 135.3 kcal/mol, respectively. The conclusion made in [69] was confirmed by the results of [71], concerning the significant effect on the stabilization of higher polynitrogens by cyclic fragments N_5_ and N_3_. N_14_ and N_16_ are also mentioned in [72], but only N_14_ (*D_3h_*) and N_16_ (*D_4h_*) with two quadrangular and eight hexagonal faces were discussed there. Octadecanitrogens N_18_ have been the study objects, apparently, only of a single study [73], although there is information about one of these compounds, N_18_ (*C_2v_*) with one hexagonal, two quadrangular, and eight pentagonal faces in [72], too. Within this publication, 18 isomeric octadecanitrogens with a three dimensional structure are described, the most stable of them is N_18_ (*D_3h_*) with two triangular, six pentagonal and three hexagonal faces, and the least stable is N_18_ with the same *D_3h_* symmetry, but formed by six quadrangular and five hexagonal faces (Scheme 15); the difference in their total energies, according to the HF, MP4//HF, and MP4//B3LYP data, is 185.4, 189.1, and 188.0 kcal/mol, respectively. No data on octadecanitrogens with planar (pentazole) and/or acyclic molecular structures has yet appeared.

**Scheme 16:**
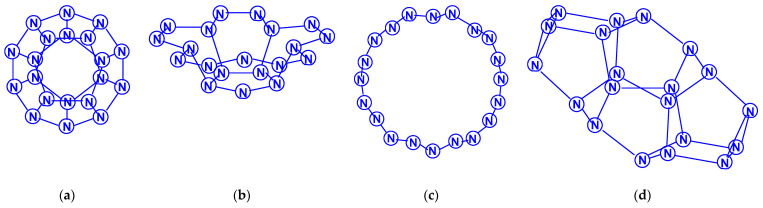
(**a**): N_20_ (*I_h_*); (**b**): N_20_ (*D_5v_*); (**c**): N_20_ (*D_5_*); (**d**): N_20_ (*C_2_*).

Eicosanitrogens N_20_ have also been considered in a small number of publications [74,75,76,77]. As early as 30 years ago, an article [74] was published: using the DZP SCF method, it was shown that N_20_ has a dodecahedral geometry with an energy of about 50 kcal/mol per ten N_2_ molecules. A more detailed study [75] of eicosanitrogen isomerism at the levels of the MP2 and B3LYP theory allow the identification of three minima on the potential energy surface of N_20_:N_20_ (*I_h_*) in the form of an icosahedral cage, N_20_ (*D_5v_*) in the shape of a bowl, and N_20_ (*D_5_*) in ring form (Scheme 16). According to the data of this work, the total energies of these structures are in the order E_total_ [N_20_ (*D_5_*)] < E_total_ [N_20_ (*D_5v_*)] < E_total_ [N_20_ (*I_h_*)], with the N_20_ (*I_h_*) having energy almost 200 kcal/mol higher than the N_20_ (*D_5_*). In [76], another isomer was added to this list, namely, N_20_ (*C_2_*) in the form of a polyhedron with two triangular, six pentagonal, and four hexagonal faces, and the parameters of this structure were compared with the analogous parameters of N_20_ (*I_h_*). It was noted that N_20_ (*C_2_*) is also a more energetically favorable structure compared to the dodecahedral structure (although by not much—only by 16–25 kcal/mol, depending on the calculation method). An attempt to explain this phenomenon is presented in the same paper [76] and is based mainly on geometric features (“Despite an apparently ideal structural environment, the N_20_ dodecahedron is not the most stable cage isomer. The cause is an energetic penalty resulting from the eclipsed atoms and lone pairs in the planar pentagons in the N_20_ dodecahedron. same reason. Also, this eclipsing effect may contribute to the instability of spheroidal isomers relative to cylindrical ones at other molecule sizes as well”), that, however, is not very convincing. Taking into account such the small number of studied N_20_ isomers (only 4), it is premature to draw any conclusions about the geometry that will be inherent in the molecular structure of the lowest energy isomer of a given composition (especially after the publication of [76]; eicosanitrogens were mentioned only in the work already cited above [72], as well as in [77] with an assortment of polynitrogens from N_6_ to N_60_ (and only about N_20_ (*I_h_*))).

### 3.3. Polynitrogens with More than 20 Nitrogen Atoms

**Scheme 17:**
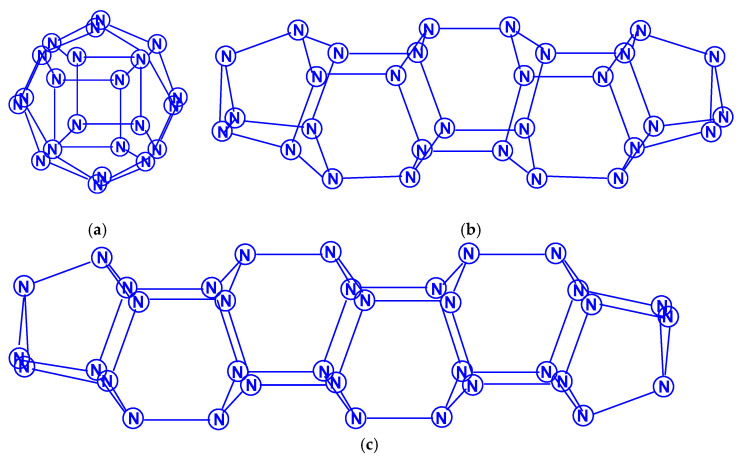
(**a**): N_24_ (*D_4h_*); (**b**): N_30_ (*D_3h_*); (**c**): N_30_ (*D_3d_*).

The polynitrogens indicated in the title (judging by the number of papers [77,78,79,80,81,82,83,84,85,86,87,88], excluding [78,81], were published in the 21st century), have attracted more attention than the polynitrogens N_12_–N_20_. The most extensive, by the number of polynitrogens studied, should be recognized as [77], in which the structural and thermodynamic parameters of a very significant number of polynitrogens with an even number of atoms, namely, N_6_ (*D_3h_*), N_8_ (*O_h_*), N_10_ (*D_5h_*), N_12_ (*D_6h_*), N_12_ (*D_3d_*), N_16_ (*D_4d_*), N_18_ (*D_3h_*), N_20_ (*I_h_*), N_24_ (*D_3d_*), N_24_ (*D_4h_*), N_24_ (*D_6d_*), N_30_ (*D_3h_*), N_30_ (*D_5h_*), N_32_ (*D_4d_*), N_36_ (*D_3d_*), N_40_ (*D_4h_*), N_42_ (*D_3h_*), N_48_ (*D_4d_*), N_48_ (*D_3d_*), N_54_ (*D_3h_*), N_56_ (*D_4h_*) and N_60_ (*D_3d_*); wherein, N_48_ (*D_3d_*), N_54_ (*D_3h_*), N_56_ (*D_4h_*), and N_60_ (*D_3d_*), which are divided into four sets, were studied in the most detail. Some of these polynitrogens are presented in Scheme 17.

**Scheme 18:**
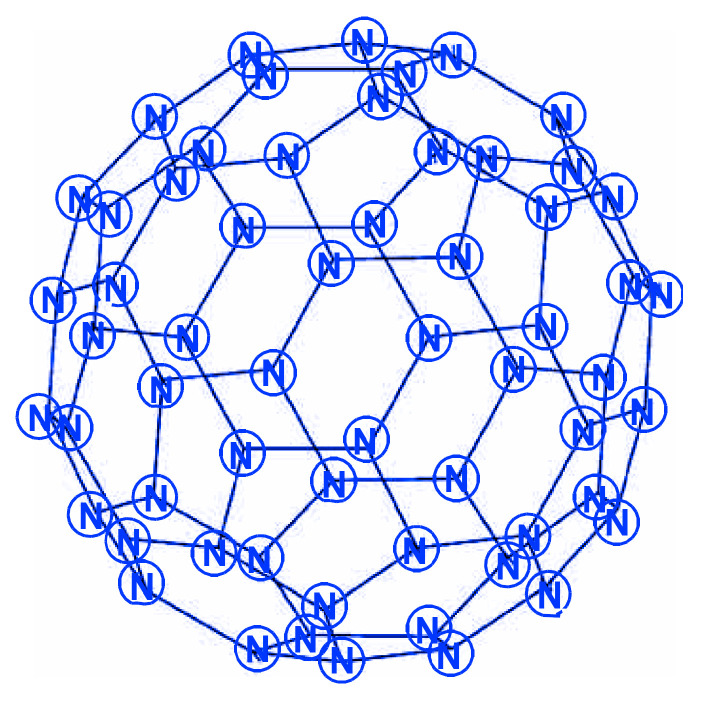
N_60_ (*I_h_*).

The molecular structures N_18_ of the N_3_(N_5_)_3_ type and N_30_ of the N_5_(N_5_)_5_ type were also considered in a later work [78]. According to these data, on the one hand, the five membered rings of nitrogen atoms play a major role in the stability of the molecule cage; in this case, the lengths of bonds, on both sides of which five membered rings are located, are the shortest. The role of three membered rings is less significant. On the other hand, for molecules of isomeric cages, the more layers in the molecules, the more stable the molecules; in this regard, the cylindrical isomers of the nitrogen cage molecules are more stable than the spherical ones. These conclusions are quite consistent with the results obtained using the levels of the HF and DFT B3LYP theory in [80], where the isomeric compounds N_24_ (*D_4h_*), N_30_ (*D_3h_*), N_30_ (*D_5h_*) and N_36_ (*D_3h_*), N_36_ (*D_4h_*) with N–N single bonds are discovered. Tetracosanitrogen N_24_ was also considered in [79] (isomer N_24_ (*D_6d_*), method B3LYP/6-31G*), and in [81], where the main emphasis was not on calculating its characteristics, but on considering a new approach to assessing stability, based on determining the shape and size of cages obtained by the partial replacement of individual nitrogen atoms by carbon atoms. In particular, in this work, three dimensional N_22_C_2_ cages obtained by replacing two nitrogen atoms in N_24_ were used for this purpose. In [59,77,82,83], a quantum chemical consideration of the N_60_ hexacontanitrogen molecule isomeric forms was carried out. The first study [82] reported on the calculation of two N_60_ molecule isomeric forms, N_60_ (*I_h_*), the image of molecular structure of which is presented in the Scheme 18, and N_60_ (*S_6_*) by the HF/STO-3G and HF/6-31G methods; the authors concluded that the second of these isomers is more stable than the first. In the article [59], the geometry was optimized and the IR active vibration frequencies N_60_ (*I_h_*) formed from N_10_ building blocks were calculated at the SCF/cc-PVDZ, SCF/6-31G*, and AM1 theory levels. Using the HF/cc-pVDZ method, the energy of the reaction N_60_ (*I_h_*) → 6N_10_ was estimated (2430 kcal per 1 mol N_60_). In this regard, it was suggested that the N60 (Ih) molecule can be formed experimentally under extreme pressure conditions. This conclusion does not seem very convincing, because of the data of works [77,82,83]: on the one hand, N_60_ (*S_6_*) is more energetically stable, and, on the other hand, N_60_ (*I_h_*) is not a kinetically inert compound. In [83], the geometry was also optimized and the vibration frequencies at the HF/6-31G* and B3LYP levels were calculated for the N_60_ (*I_h_*) and N_60_ (*S_6_*) structures; it turned out that the molecular structure of symmetry Ih is not a minimum on the potential energy surface N_60_ (the calculation showed the presence of four imaginary frequencies at the HF/6-31G* level and 12 imaginary frequencies at the DFT B3LYP/6-31G* level), while N_60_ (*S_6_*) symmetry at the HF/6-31G* level revealed two structures with corresponding minima, and one minimum for the *S_6_* symmetry structure at the B3LYP/6-31G* level. The difference between the HF and B3LYP predictions was explained by the absence of dynamic electron correlations in the HF method. Even more complex polynitrogens, N_66_, N_72_, N_78_ and N_84_, are described in [84,85,86,87], respectively. All these works were carried out by the same research group, and, therefore, in each of them the same analysis methodology and set of calculation methods were used: the structure and energies were studied at the B3LYP/cc-pVDZ level. In addition, one point energy calculations were completed at the MP2/cc-pVDZ and B3LYP/cc-pVTZ levels, to determine the presence of dispersion interactions. In each of these works, only one compound was investigated, namely, N_66_ (*D_3h_*), N_72_ (*D_3h_*), N_78_ (*D_3h_*), and N_84_ (*D_3h_*) with a prismatic form. All of these compounds belong to the group of polynitrogens with the general formula (N_6_)_n_ (n = 1–35, with alternative symmetry *D_3h_* or *D_3d_*) and are the eleventh, twelfth and thirteenth members of this series, respectively, that can form various nanotubes [88]. In this work, as well as in [84,85,86,87], the MP2/cc-pVDZ and B3LYP/cc-pVTZ methods were used for calculations. It has been established that van der Waals forces or dispersion interactions are the dominant stabilizing factor for large prismatic nitrogen cages. The van der Waals forces intertwine with the covalent bonds to form a dense network that tightly binds the nitrogen atoms at the network nodes and makes the (N_6_)_n_ type prismatic molecules stable. It is expected that this will open up great prospects for the study and application of nitrogen nanofibers, because, according to [88], nitrogen-containing nanotubes should be environmentally friendly. Publications of the last decade [89,90] concerned the problems of the topology of polynitrogens, from tetranitrogen N_4_ to eicosahectanitrogen N_120_.

Section 3 shows that the most diverse polynitrogens with an even number of atoms and geometric structures are theoretically quite admissible. At the same time, for a small number of atoms (k ≤ 8), apparently, the results of calculations obtained by various versions of the DFT method, agree quite well with the results of calculations using higher level methods (MP4, CCSD, etc.), at least measuring by quality. With an increase in the atoms number (k) in polynitrogen molecules, naturally, the number of possible molecular structures, and primarily three dimensional structures, also increases, and different theoretical methods give different (and often quite contradictory) results. It is also obvious that far from all possible structures have been identified for polynitrogens with k > 10 (and their relative stability, based on the data that the most stable form is determined for each individual N_k_, cannot be considered unequivocally established either), and new, larger scale studies are absolutely necessary.

## 4. Neutral Polynitrogens N_k_ Containing an Odd Number of Atoms

**Scheme 19:**
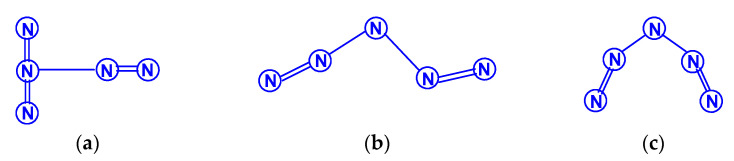
(**a**): N_5_ (*C_2v_*); (**b**): N_5_ (*C_s_*); (**c**): N_5_ (*C_2v_*).

From the standpoint of classical concepts, each nitrogen atom is connected to other atoms by exactly three (no more and no less) chemical bonds by the exchange mechanism, and the number of nitrogen atoms in stable neutral polynitrogens should be an even number. In neutral polynitrogens with an odd number of atoms, at least one nitrogen atom must be bonded to its neighbors by either two or four of the above chemical bonds. In the literature, there are separate (albeit purely theoretical) data on polynitrogens with an odd number of atoms, but, in none of these compounds does that which has just been said take place. The simplest of them are N_3_ trinitrogens, which are no longer of particular interest among researchers of the physico-chemistry of polynitrogens, and therefore we will not specifically focus on them in this review; information about earlier works on this compound can be found in the review mentioned above [9]. In general, in addition to N_3_, some works consider polynitrogens with an odd number of atoms from 5 to 15 [38,59,78,91,92,93,94,95,96,97,98,99,100,101,102]. The next most complex category of polynitrogens with an odd number of nitrogen atoms, N_5_, is the subject of works [38,59,78,91]. According to the article [91], the most stable form is predicted to be (N_2_)(N_3_) with a binding energy of 1.2 kcal/mol, while the pentazene structure is energetically less favorable by 40.6 kcal/mol and the open structure of N_5_ (*C_2v_*) by 56,16 kcal/mol (Scheme 19). The formation heats were also estimated for normal conditions and amounted to (179 ± 2.4) kcal/mol [91]. All studies agree that the potential well for N_5_ is very shallow, making it rather unstable. In the work [59], the geometry of the closed ring N_5_ was optimized using the MP2, B3LYP, and QCISD methods; at the same time, it was noted that, using the MP2 method, such optimization led to the “breaking” of the N_5_ cycle, and the calculations of the second derivatives at all levels proved that the optimized structure is a first order saddle point leading to further decomposition up to N_2_. N_5_ as an intermediate product of hexanitrogen degradation was discovered in [38], as a result, it was stated that such a molecule does not exist as a discrete species. Thus, the conclusion drawn in [91], about the low stability of pentanitrogens, was confirmed. It was shown in [78] that all energetically preferable structures appear with an angle to angle connection between two rings for cyclic N_3_ and/or cyclic N_5_.

**Scheme 20:**
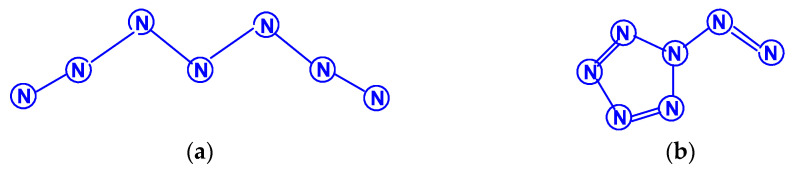
(**a**): N_7_ (*C_2v_*); (**b**): N_7_ (*C_s_*).

For the neutral heptanitrogen N_7_, seven isomers were considered in [92] at the theoretical levels UHF/6-31G*, UMP2/6-31G*, and B3LYP/6-31G*. It was found that five of them correspond to minima on the potential energy surface (four with *C_s_* symmetry and one with *C_2_* symmetry), among them there is a one cap “open book”, a structure in which two triazene rings are connected to each other through a nitrogen atom; a “capped” distorted hexagon; and two twisted acyclic structures *C_2_* or *C_s_* with an open chain, and these are the most energetically favorable. Later, the authors of [93] reported calculations at the levels of the UHF, MP2, and B3LYP theories of five more isomers, namely, in the form of a bath; an articulated quadrilateral and pentagon; linear N(N_3_)_2_, with a pentazole ring and a side chain N_2_ (N_2_)(N_5_) (Scheme 20); and a capped hexagon, in which the “capped” nitrogen atom is connected by three bonds to the nitrogen atoms of the cyclic fragment. According to this data presented, the acyclic W shaped isomer is the most stable, in good agreement with the conclusion drawn in [92]. However, this does not coincide with the data presented by the same group of researchers in [94], where the structure with a pentazole ring and an N_2_ side chain turned out to be the most energetically favorable. In the same work, it was also found that the stability of some of the stable isomers is enhanced by conjugation or hyperconjugation. Perhaps further theoretical studies of the structures of heptanitrogens will be able to reveal even more stable N_7_ isomers, but this question still remains open, especially since, in recent years, no new publications on heptanitrogens have appeared.

**Scheme 21:**
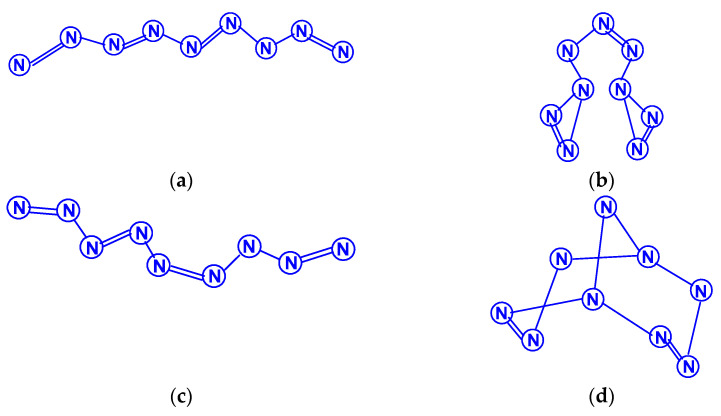
(**a**): N_9_ (*C_2v_*); (**b**): N_9_ (*C_2v_*); (**c**): N_9_ (*C_s_*); (**d**): N_9_ (*C_2v_*).

In the article [95], using the HF, MP2, and DFT methods, the calculation of stable molecular structures for the nonanitrogen N_9_, as well as the N_9_^+^ cations and N_9_^−^ anions formed by it, was performed. Four minima were revealed on the potential energy surface of N_9_, and the structures, energies, and vibrational frequencies of the corresponding N_9_ isomers were calculated. The images of the molecular structures of these isomers are shown in Scheme 21. According to this work, the N_9_ (*C_2v_*) isomer is the most stable. Possible pathways of N_9_ (*C_2v_*) dissociation were detected using the HF/6-31G*, B3PW91/6-31G*, B3LYP/6-31G*, and MP2/6-31G* methods [96]. The transition states of decomposition processes on the potential energy surfaces HF/6-31G*, B3PW91/6-31G*, and B3LYP/6-31G* are discovered and characterized, and the energy barriers of the N_9_ → N_6_ + N_3_ process are calculated, that with corrections for zero energy are predicted to be 15.3, 31.8 and 32.9 kcal/mol, for the N_6_ → 3N_2_ process (second stage of decomposition)–8.2, 16.3 and 14.4 kcal/mol, respectively. The N_9_^+^ → N_7_^+^ + N_2_ barrier height is 2.1 kcal/mol, and the second stage N_7_^+^ → N_2_ + N_5_^+^ is 4.3 kcal/mol at the B3LYP/6-311+G*//B3LYP/6-31G* level. The authors of [97] calculated the energy barriers for the dissociation of a number of N_9_ isomers (as well as N_11_) at the HF, MP2, and CCSD(T) theoretical levels. In this work, only acyclic zigzag structures were analyzed, each of them containing two practically linear N_3_ groups and three (N_9_) or five (N_11_) zigzag binding nitrogen atoms. A total of four N_9_ structures (three with *C_s_* symmetry, one with *C_2v_* symmetry) and five N_11_ structures (4 with *C_s_*, 1 with *C_2v_* symmetry) were studied. The energy barriers of dissociation reactions (depending on the isomer and calculation method, varies in the range of 10–30 kcal/mol) are probably too high to try to register even the most stable of them in the experiment.

**Scheme 22:**
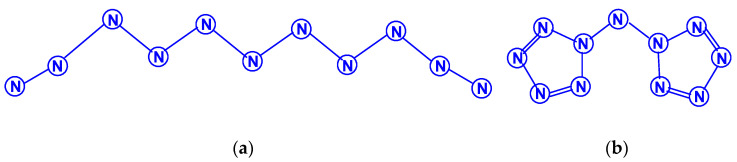
(**a**): N_11_ (*C_2v_*); (**b**): N_11_ (*C_2v_*).

The molecular structures of undecanitrogens, also in [96], were considered in [98,99,100]. Stable structures for N_11_, N_11_+, and N_11_^−^ were calculated using HF, MP2, CCSD(T), B3LYP, and B3PW91 theoretical levels [98]. As follows from these calculations, the most favorable structure in the case of N_11_ and N_11_^−^ is the acyclic N_5_(N_3_)_2_, which is composed of two almost linear N_3_ fragments interconnected by a zigzag N_5_ grouping (Scheme 22). The dissociation and isomerization reactions of N_11_ isomers, including two structures previously studied in [98], as well as three new structures, were investigated using three variants of the DFT method, namely, DFT B3LYP/6-31G(d), DFT B3LYP/6-311G(d) and DFT B3LYP/6-311+G(3df)//B3LYP/6-311G(d) theory levels in [99]. It was shown, as in the previous results for the N_9_ and N_10_ isomers, that the barrier height for the structures described in this work in the case of the removal of N_2_ is about 10–15 kcal/mol, while the barrier height in the case of removal of N_3_ is 25–30 kcal/mol. Therefore, N_2_ appears to be easier to remove than N_3_ from relatively large nitrogen isomers. In addition, for each of these three structures, both dissociation and isomerization can occur; moreover, isomerization seems to be preferable even to dissociation, due to its relatively low energy barrier height. Equilibrium geometries, energies, and vibrational frequencies for eight low spin N_11_ isomers were calculated [100] at the HF/6-31G*, DFT B3PW91/6-31G*, DFT B3LYP/6-31G*, and MP2/6-31G* levels of the theory. According to this work, the pentazole ring remains the fundamental stable structural unit of the molecular structure of N_11_ polynitrogens. In this case, the most stable N_11_ isomer in terms of energy is N_11_ (*C_2_*), which consists of two aromatic pentazole rings connected to each other through a bridging nitrogen atom (Scheme 21). However, the study of its decomposition reaction shows that this isomer has a low energy barrier for its decomposition reaction (only 5.6 kcal/mol according to DFT B3LYP/6-31G*); so, it seems that this structure is a kinetically unstable species with an open shell.

**Scheme 23:**
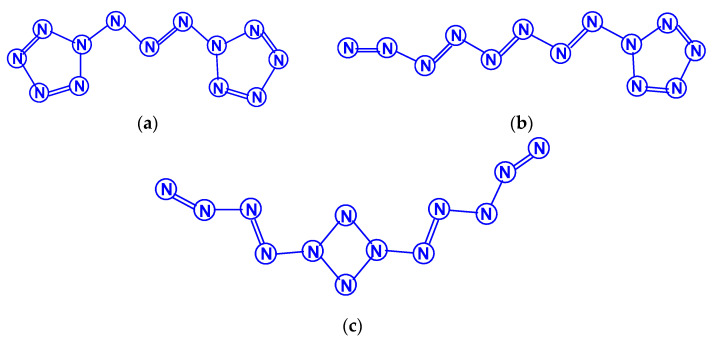
(**a**): N_13_ (*C_2v_*); (**b**): N_13_ (*C_s_*); (**c**): N_13_ (*C_s_*).

Neutral tridecanitrogens N_13_ were detected only in [101], where a quantum chemical study of nine low spin N_13_ isomers was carried out at the theoretical levels UHF/6-31G*, UB3LYP/6-31G*, and UMP2/6-311G*. The most stable isomer was the N_13_ (*C_2v_*), which contains two separate pentazole rings connected by three nitrogen atoms (Scheme 23). The structures containing pentazole rings were generally more stable than the zigzag acyclic structure and those containing four and six membered rings. The authors of the given work [101] believe that the pentazole ring is a fundamentally stable structural unit applicable not only to polynitrogens with even numbers of atoms but also to polynitrogens with large odd numbers of nitrogen atoms. However, in our opinion, there are still insufficient grounds for such a conclusion given the very poor knowledge of tridecanitrogens.

**Scheme 24:**
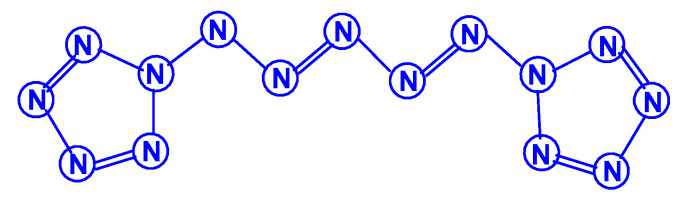
N_15_ (*C_2v_*).

Information on neutral pentadecanitrogens N_15_ is also contained in only one work [102], in which, in addition to N_15_, polynitrogens with smaller odd numbers of nitrogen atoms, N_11_ and N_13_, were also considered. The possible structures of N_15_ were investigated by the MP2 and DFT B3LYP/6-31G* methods. According to this data, for polynitrogens N_k_ with even k = 8–14, as well as for polynitrogens with odd k = 11–15, all the thermodynamically most stable isomers are based on pentazole units; moreover, for each of them, the more conjugated the five membered rings, the more stable the isomer (Scheme 24) and the more side chains that link such cycles, the less stable it is. On the whole, polynitrogens with an even number of nitrogen atoms, as expected, are more energetically stable than polynitrogens molecules containing an odd number of nitrogen atoms [102]. Concluding Section 4, we note that no sufficiently reliable experimental data confirming the results of the above mentioned theoretical works [38,59,78,91,92,93,94,95,96,97,98,99,100,101,102] have been obtained so far.

## 5. Oligomeric/Polymeric Nitrogen Compounds

This variety in polynitrogens was the subject of publications [103,104,105,106,107,108,109,110,111,112,113,114,115,116,117,118]. For example, in [103], a polymeric form of nitrogen was reported, where all atoms are linked by single covalent bonds, similar to carbon atoms in a diamond; this compound was synthesized directly from molecular dinitrogen at temperatures above 2000 K and pressures above 110 GPa using a laser heated diamond cell. It was identified by X-ray spectroscopy and the Raman scattering of polymer nitrogen to have a theoretically predicted cubic gauche structure (cg-N). In article [104], another synthesis variant of this allotrope was proposed by using a mixture of nitrogen and argon flowing through bulk sodium azide or sodium azide dispersed on multiwalled carbon nanotubes 100 nm long, when the reaction system is exposed to radiofrequency plasma at temperatures close to ambient temperature. This allotropic modification of nitrogen is a metastable compound; it has an energy capacity more than five times higher than the capacity of the most energy intensive materials. The given modification of polymer nitrogen was also discovered in [105,106], where its electronic structure was calculated using the density functional theory; exchange correlation functionals, including the local spin density approximation; the generalized gradient approximation (GGA); the ab initio meta-GGA; and the hybrid functional shielded exchange. It has been shown that cubic polymeric nitrogen has an even higher energy storage capacity than reported in [103]. The possibility of the existence of other forms of polymeric nitrogen containing only single N–N bonds was also noted: layered, existing in the pressure range P = 188–320 GPa [107,108]; helical tunnelling, existing at P > 320 GPa [107]; sheet zigzag [109,110]; trigonal [111]; and even metal [112,113,114] (although it is very difficult to assume that nitrogen atoms, due to their high electronegativity, can participate in the formation of metal bonds!). The existence of polymeric nitrogen, having a “parquet” structure and composed of N6 hexagons, and resembling the structure of black phosphorus or graphite [115], and also in the form of nanotubes [116], is possible. Finally, in the most recent work [117], a new class of polymeric nitrogen molecules was investigated, consisting of several intergrown nanotubes of nanometer length—astralenes, which, depending on the shape of the central part (core), can be divided into cubic, hexagonal, and tetrahedral. Using the DFT method, it was shown that astralenes can be used to form covalent crystals—new allotropic forms of nitrogen with Pm3m, P6/m, and Fd3m symmetry and are semiconductors with an energy gap in the range from 0.32 eV to 2.04 eV. A critical review of the synthesis and prediction perspectives for the existence of various polynuclear nitrogen compounds was presented in the article [118].

## 6. Possibilities of Practical Application of Polynitrogens

In many of the works mentioned above, [22,23,64,95,97,103], and, also, in [119,120,121,122,123,124,125,126,127,128], the question of the possibility of using polynitrogens in various aspects was analyzed, mainly as potential “green” explosives, because the process of their decomposition is associated with a significant increase in entropy and is accompanied by the formation of only molecular nitrogen (dianitrogen) N_2_; in this case, polynitrogens with both an even (as, for example, in [64]) and an odd (as, for example, in [95]) number of nitrogen atoms in the molecular structure were considered in this capacity. It is interesting to note the article [124], which systematizes experimental data on the physicochemical parameters of 300 high energy compounds from different classes (enthalpies of formation, densities of molecular crystals, detonation parameters, and sensitivity to various types of impacts) and develops calculation schemes for evaluating these characteristics. For polynitrogenous compounds, the high efficiency of polynitrogens such as octaazacubane, octaazatetraene, etc., has been predicted. The energetic properties of both individual compounds and compositions containing these substances, together with others, were evaluated in order to assess the effectiveness of their use as components of solid composite fuels. In addition to this possibility, attention was drawn to one more aspect of their use, namely, to the reactions of oxidation with molecular oxygen, i.e., as combustibles [22,23]. This possibility was demonstrated on the example of the polynitrogens N_4_ (*T_d_*), N_4_ (*D_2h_*), N_6_ (*C_2v_*) and N_8_ (*O_h_*); on the basis of the thermodynamic parameters of the reactions of their oxidation to nitrogen monoxide NO (Δ_r_*H*^0^, _r_*S*^0^ и Δ_r_*G*^0^) calculated by the G4 method. It was noted that all these reactions are exothermic and irreversible, which sharply distinguishes them from a similar reaction to the interaction of dinitrogen N_2_ with oxygen, which is accompanied by the formation of NO (which is known to be endothermic and can only be realized at very high temperatures). The article [104] also focused on the possibility of using polynitrogens as catalysts for various chemical reactions.

The applications of polynitrogens are very important, but even more important is the possible use of polynitrogens in rocket technology, where such materials with a high enthalpy of formation are required (which is very important in solving problems aimed at increasing the speed and flight range of a rocket), and the high energy density of the initial substance (to increase the share of the payload while maintaining the total mass of the rocket unchanged). In the 21st century, these materials received a special term “high energy density material” (HEDM), which has become widely used in the world literature to designate this type of energy materials [123]. According to theoretical (as well as still few experimental) data, polynitrogens are characterized by uniquely high enthalpies of formation per unit mass (from 2000 to 5000 kcal/kg) and a fairly high density in the condensed phase (2–4 g/cm^3^) [120]. For this reason, the use of polynitrogens will make it possible in the future to create a new generation of solid rocket fuels that can successfully compete with liquid rocket fuels in terms of energy efficiency. However, even more importantly, their use changes the principle according to which a working fluid is formed in a rocket engine—the so called high enthalpy gas. In the classical scheme of its formation adopted in modern rocket technology as a result of the oxidation of rocket fuel (hydrocarbons, 1,1-dimethylhydrazine (“heptyl”), etc.) with oxygen or another oxidizing agent, the thermal energy released as a result of the reaction is spent on heating the reaction products. Using polynitrogens, their decomposition itself leads to the formation of hot molecular dinitrogen, which creates thrust when it exits the nozzle. In addition, probably, the breakdown of polynitrogens will provide higher values of specific impulse (Isp) than oxidation reactions [120]. Another advantage of using polynitrogens as rocket fuel is that their decomposition produces dinitrogen, which does not lead to environment pollution (whereas such pollution always occurs in the case of traditional rocket fuels). Review articles related to the use of polynitrogens as HEDM have also been published [122,123,126,127,128].

## 7. Conclusions

Thus, based on the available calculated data, it can be argued that the existence of polynitrogens with a very significant (more than 100) number of nitrogen atoms in the molecular structure is quite possible. With an increase in the number of atoms in polynitrogen molecules, the complexity of calculations and the time spent on their implementation increase significantly; therefore, virtually all available data on the structural and energy characteristics of polynitrogens N_k_ with k > 20 were obtained either by the DFT method or by methods of a lower level. Nevertheless, a comparison of the quantum chemical calculations at various levels (HF, DFT, MP, CCSD etc.) for a wide variety of polynitrogens presented in the cited publications allows us to assert that, in a qualitative sense, these results, as a rule, are in good agreement with each other. Due to the predictions of all these methods, among the set of N_k_ isomers with the same value of k (regardless of whether k is even or odd), the isomer with the lowest level of symmetry almost always turns out to be the most stable from the energy point of view. However, this seems to be quite understandable if we take into account the fact that, in nature, the order is a less probable category than disorder, because of an increase in the degree of order in any physicochemical system in general and molecules in particular (which is accompanied by a decrease in entropy) certain energy costs are required and occur only if the formation of new, stronger chemical bonds occurs. As for quantitative data (the parameters of molecular structures, distribution of electron density, binding energies between nitrogen atoms, total energies of molecules, their dissociation energies, standard thermodynamic parameters of formation, etc.), we can only talk about a fairly good agreement between the parameters of molecular structures (bond lengths, bond and dihedral angles) calculated by various methods. For the other indicators listed above, discrepancies between the corresponding digital data obtained by various methods, are sometimes quite significant. However, such a difference should not be embarrassing, because, at the current state of mathematical physics and the theory of molecular structure, it is still impossible to consider all possible types of interatomic and intermolecular interactions that take place even in the simplest molecules. In addition, therefore, in each of the used quantum chemical methods, there only some of these interactions can be taken into account. The situation is further complicated by the absence of any experimental data of the polynitrogens’ physicochemistry, that, unfortunately, does not allow today the verification of the reliability of the calculated data available in the literature for these unique compounds. As more and more powerful computer systems and technologies become available, and theoretical possibilities become more and more perfect, there is every reason to believe that the reliability of the calculated data will also increase and, eventually, they will be fully consistent with the corresponding experimental data.

Summing up, a paradoxical situation has arisen on the issue of polynitrogens: there is so much calculated data on polynitrogens at the current time that it is very difficult to combine them into any single story, while there is little reliable experimental data. Today, there are a lot of abstracts in this specific area of chemical science, but very little concrete experimental data for concrete polynitogenes. In our opinion, there is all reason to hope that the difference between the number of theoretical and experimental works on polynitrogens will steadily decrease over time, and, in the future, many of the chemical nitrogen compounds mentioned above will be obtained and used in anthropogenic activities.

## Data Availability

No unpublished data was created or analyzed in this article.
